# Isokinetic Strength and Functional Scores after Rehabilitation in Jiu-Jitsu Fighter with Repair Surgery of Pectoralis Major Muscle Rupture: A Case Report

**DOI:** 10.3390/healthcare9050527

**Published:** 2021-04-30

**Authors:** Guangyi Hu, Quan Jiang, Ji-Young Lee, Yong-Hwan Kim, Duk-Han Ko

**Affiliations:** 1 Department of Physical Education, General Graduate School, Yongin University, Yongin 17092, Korea; huguangyi84@163.com; 2 Department of Physical Education, Gangneung-Wonju National University, Gangneung 25457, Korea; 584024910@gwnu.ac.kr (Q.J.); jylee@gwnu.ac.kr (J.-Y.L.); 3 Department of Sports Science Convergence, Dongguk University, Seoul 04620, Korea

**Keywords:** rehabilitation, pectoralis muscle, rupture, surgery, muscle strength, case report

## Abstract

A pectoralis major muscle rupture is a rare injury that mainly occurs during exercise. This study examined the application of rehabilitation, strength and passive range of motion (ROM) change, and subjective assessment for 1 year undertaken after repair surgery of pectoralis major muscle rupture in a Jiu-Jitsu fighter. We hypothesized that the application of ROM exercises and rehabilitation strategies contributed to muscle recovery and successful return to sports. The patient was a 34-year-old man who was injured after falling during a competitive event. The patient had pain and swelling in the front of the chest and shoulder, and the distal chest was deformed. Imaging revealed a complete rupture of the pectoralis major muscle. Reparative surgery was performed by a specialist. Immobilization was performed one week after the surgery. Passive ROM exercises began with the forward flexion 2 weeks after the surgery; abduction and external rotation ROM exercises at 4 weeks; low-intensity muscle strength exercises using tube bands at 6 weeks; machine-based pectoralis major muscle exercises at 3 months. Isokinetic equipment was used to measure horizontal adduction and internal rotation strengths, and the subjective shoulder functional and ROM scores were evaluated. Recovery of shoulder function and ROM occurred at 3 months and muscle recovery at 6 months. The participant was able to return to sports at 5 months and compete at 7 months. Although this study explored only one patient’s post-operative recovery, it suggests that ROM and strength exercises may be effective post-operative strategies for restoring function and strength to enable a return to sports.

## 1. Introduction

Pectoralis major (PM) muscle rupture is a rare injury mainly caused by traumatic accidents [1]. The PM muscle is one of the largest muscles in the anterior part of the upper body. It is primarily moved by the humerus. When complete rupture occurs, pain and discomfort are often present during when trying to perform the PM’s major functions, namely, adduction, internal rotation, and flexion of the humerus [2,3].

The injury mechanism involves the extension and external rotation of the upper arm with high energy, and typical signs and symptoms after injury include ecchymosis, swelling, and pain. Asymmetric deformation of the anterior axillary fold can confirm the rupture [1,4]. The most noticeable characteristic of the injury is that it tends to occur during athletic or sports activities. A meta-study of 40 cases revealed that almost half occurred due to bench press or weight lifting exercises [5].

Typical medical treatments after PM rupture have non-surgical or surgical treatment. Non-surgical treatment is used when a patient presents with minor injury or low levels of physical activity such as elderly [1,6]. Meanwhile, surgery is used when it is necessary to recover PM function [7]. A previous study reported that surgical treatment enabled patients with PM rupture to recover up to 99% of peak torque levels, while non-surgical treatments enabled patients to recover only up to 56% of peak torque levels (non-surgical patients were still generally able to participate in sports activities and daily life) [7]. A case report of the time taken to return comfortably to daily activities after surgery reported that showering and bathing were performed at 2.5 weeks; dressing using both arms at 4 weeks; swimming at 10 weeks; contact sports, such as basketball and volleyball, at 16 weeks [8].

Although PM muscle rupture usually occurs during sports and rehabilitation is important for a successful return to sports activity after surgery, few studies address PM muscle rehabilitation. Instead, most existing literature presents only case reports of exercises, such as the pendulum exercise, performed over one month after reparative surgery [8,9,10]. Although this study is also a case report, it contributes to existing literature by offering the impact of rehabilitative strategies performed more shortly after surgery: early rehabilitation was initiated by applying tolerated passive range of motion (ROM) exercises 2 weeks after surgery. While existing literature on rehabilitation protocols advises beginning passive ROM exercises 2 weeks after surgery after 1 week of immobilization [11], previous case reports only offer the results of beginning the pendulum exercise at 5 weeks post-surgery after 4 weeks of immobilization, forward flexion at 6 weeks post-surgery [8], active assist ROM exercises at 5 weeks post-surgery, and passive ROM exercises at 12 weeks post-surgery [9].

The patient in the current case study was an amateur Jiu-Jitsu fighter. Jiu-Jitsu is a physically demanding sport and a technique used by mixed martial arts athletes; more specifically, it combines striking, twisting, and knocking down an opponent. Since Jiu-Jitsu involves many techniques using the arms, fighters often experience higher injury rates in the upper body than in the lower body [12]. Finally, in this study, isokinetic strength, ROM, and shoulder function scores were measured and evaluated for one year during rehabilitation. We hypothesized that the early application of passive ROM exercises contributed to the patient’s muscle recovery and successful return to sports.

## 2. Materials and Methods

### 2.1. Patient Information

The patient was a 34-year-old amateur Jiu-Jitsu fighter who fell backwards during a match, put his left arm on the ground, and became tangled with a competitor. Primary concerns and symptoms of the patient were pain (visual analog scale, VAS 7) in the chest and anterior shoulder area after the injury, along with swelling, and feeling discomfort when lifting or rotating arm. Moreover, a deformity of the distal chest was found. The patient had no previous history of shoulder and chest injury and underwent posterior cruciate ligament reconstruction of right knee 6 years ago. There were no other special psychiatric and psychological discomforts.

### 2.2. Clinical Finding and Diagnostic Assessment

**Figure 1:**
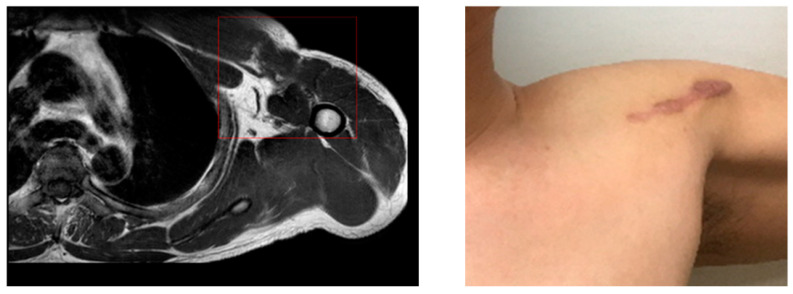
Patient’s MRI and injury site.

After a magnetic resonance imaging (MRI) at a local hospital, it was advised that the patient be moved to a superior hospital for surgery (Figure 1).

Seven days later, the MRI was read by a radiologist; the final diagnosis was a complete rupture of the pectoralis major muscle. In a physical examination conducted by an orthopedic surgeon, active forward flexion ROM was almost 160 degrees; however, external rotation was passively possible to 30 degrees, and internal rotation was limited at 35 degrees. The patient complained of pain and considerable discomfort during the examination.

The orthopedic surgeon recommended repair to the PM muscle, and the patient agreed. The patient underwent a heart examination before surgery and was treated by an internal medicine specialist and anesthesiologist. Surgery occurred 15 days after the initial injury.

The patient provided written informed consent about surgical treatment, rehabilitation, measurement, and use of the results for research. The study was approved by the researcher’s institutional review board center (approved number: GWNU IRB 2020-16) and conducted in accordance with the Helsinki Declaration.

### 2.3. Therapeutic Intervention: Surgical Treatment

**Figure 2:**
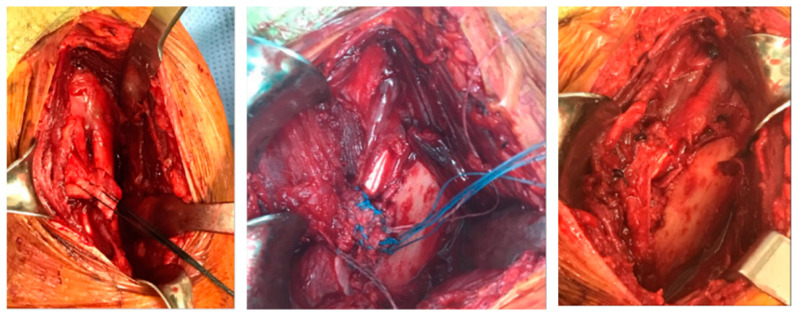
Surgical process.

The operation was performed with the patient in the beach chair position, and the approach was done in the deltoid–PM junction. First, the surgery was performed by releasing the surrounding scar tissue and connecting the ruptured area using a knot on the anchor. After the last knot, the shoulder motion and the repaired section were checked, and the skin was sutured after confirmation that there was no abnormality. The patient was discharged after 5 days with a sling (Figure 2). The patient was referred to a sports medicine center after discharge, supervised rehabilitation was conducted for 6 months, and an examination was conducted after one year.

### 2.4. Therapeutic Intervention: Rehabilitation Exercise Program

**Figure 3:**
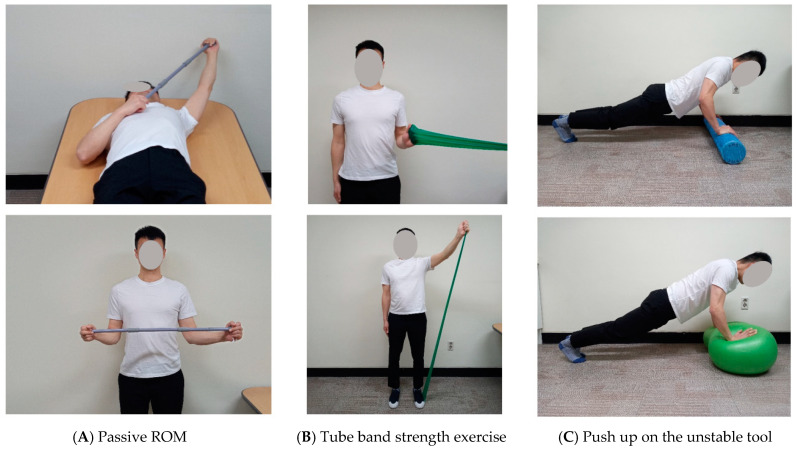
Rehabilitation exercise. (**A**) Flexion and external rotation passive stretching using T-bar; (**B**) internal rotation and flexion strength exercise using tube band; (**C**) neuromuscular training using foam roller and gym ball.

The rehabilitation program was developed with reference to existing literature [10,11] and was regularly confirmed by a surgeon (Table 1 and Figure 3). The first week after surgery involved absolute immobilization to protect the wound and the surgical site.

Early passive ROM exercises started 2 weeks after surgery. The patient continued the Week 1 exercise and started a very mild passive ROM exercise. The patient was allowed to progressively perform forward flexion movements using his healthy hand up to a maximum of a 90° angle to shoulder level whilst in a supine position. The patient also performed the pendulum exercise, hand grip, and shoulder shrugging. The patient was instructed to be careful about pain and swelling, defined as a 4 on the visual analog scale, and was limited to tolerable exercise. Additionally, scapular exercise and elbow flexion–extension were performed. External rotation and abduction movements were absolutely prohibited.

From Weeks 4–5, forward flexion was gradually increased using a pulley or T-bar, and mild abduction and external rotation were initiated. Isometric contractions were added in the pain-free range. Multi-direction pendulum exercises were allowed. After Week 6, active-assist ROM exercises were introduced, with the goal of gradually reaching full ROM. Subsequently, low-intensity isotonic strength exercises using tubing bands (Theraband Co., Akron, OH, USA) were also introduced. The patient was instructed to progress by one color a month over six months, from yellow through red, green, blue, black, and grey. Changes in the color of the tubing band and progression of the rehabilitation phase took place after a sports medicine doctor checked the patient’s 0 to 10 visual analog scale (to confirm that it was under 4), symptoms, and repair site safety.

Weight training was resumed after 12 weeks. Chest presses were first performed using the lowest weight and a small range of motion. The patient performed internal rotation and adduction strength exercises using the tube bands. Additionally, the patient trained deltoid and bicep muscles using machines.

After 5 months, the patient began performing bench press and push-up exercises with foam rollers. After 6 months, power training was introduced to prepare for the patient’s return to sports, and high-level strength training, such as plyometric training with a medicine-ball and trampoline exercises, were also performed. For 6 months, these exercises were performed at the center 1–2 days a week and conducted locally the rest of the week. At 9 and 12 months, the patient visited the center for examination. Exercises were gradually modified and increased by mobile counseling until 12 months post-surgery.

Rehabilitation program composition and exercise training were conducted by an exercise specialist with qualifications from the American College of Sports Medicine [13]. The sports medicine doctor evaluated the rehabilitation program and decided to proceed to the stage of rehabilitation. The doctor who performed the repair surgery evaluated the overall condition of the patient and decided on treatment termination.

### 2.5. Measurement: Isokinetic Strength

The isokinetic strength tests were internal rotation–external rotation and horizontal adduction–abduction at 3, 6, 9, and 12 months. The horizontal adduction and internal rotation muscle strengths related to the PM muscle were analyzed. Strength was measured using a CSMi isokinetic dynamometer (CSMi HUMAC NORM, Stoughton, MA, USA). The tests were performed based on the current leading manual and literature [14,15].

Before examination, the patient performed warm-up exercises. The examiner provided explanations and demonstrations and then performed exercises to familiarize the patient with the patient’s execution. The patient practiced in the order of high speed, medium speed, and low speed to become familiar with the machine and repeated the exercises several times. Peak torque (Nm) and deficit were used for analysis; deficit (%) = [(un-involved side − involved side)/un-involved side] × 100.

The test contraction mode was concentric contraction, and angular velocity was 60°/s. The test was first performed on the un-injured side, and the second test on the injured side. Sufficient rest was provided between tests to maximize muscle strength. For accurate testing, the patient was instructed not to move other body parts, which were further immobilized by pads and belts.

For the adduction test, the patient sat on a chair and leaned against its back. The patient’s pelvis was fixed with a belt, and the adapter was held with the test hand, with the handrail held in the opposite hand. The test angle was measured from 10–160°. Horizontal adduction, internal rotation, and flexion were measured with the patient in the supine position. The chest and pelvis were fixed with a belt and pad. The test angle was from the neutral position to 90° for horizontal adduction, and from the neutral position to 160° for the flexion. Internal rotation was performed at 90° abduction and a 90° elbow flexion position. The test angle was measured from an external rotation of 90° to an internal rotation of 60°.

### 2.6. Measurement: Range of Motion

The ROM test measured abduction and external rotation using an electronic goniometer (CYBEX EDI 320, CYBEX Inc., Stoughton, MA, USA) at 3 weeks, 6 weeks, 3 months, and 6 months. The patient was seated in a chair and the measurement sensor was placed on the forearm. The examiner set the neutral position to 0 and asked the patient to perform an active ROM within the pain-free range. It was measured carefully so that posture was undisturbed.

### 2.7. Measurement: Shoulder Pain and Disability Index

The shoulder pain and disability index (SPADI) was used to evaluate pain and functionality at 3 and 6 weeks and at 3 and 6 months. This questionnaire contains items related to shoulder pain, function, and disability; it was adopted because PM contractions occur with the movement of the humerus [16]. The questionnaire consists of five pain questions and eight questions related to daily function or disability, with no pain or disability defined as 0, and 10 indicating maximum pain or disability. Lower scores indicate better shoulder pain and function. The formula is [(score/130)] × 100].

## 3. Results and Outcome

The patient gradually recovered ROM and muscle strength and returned to sports and competition at 5 and 7 months, respectively.

**Figure 4:**
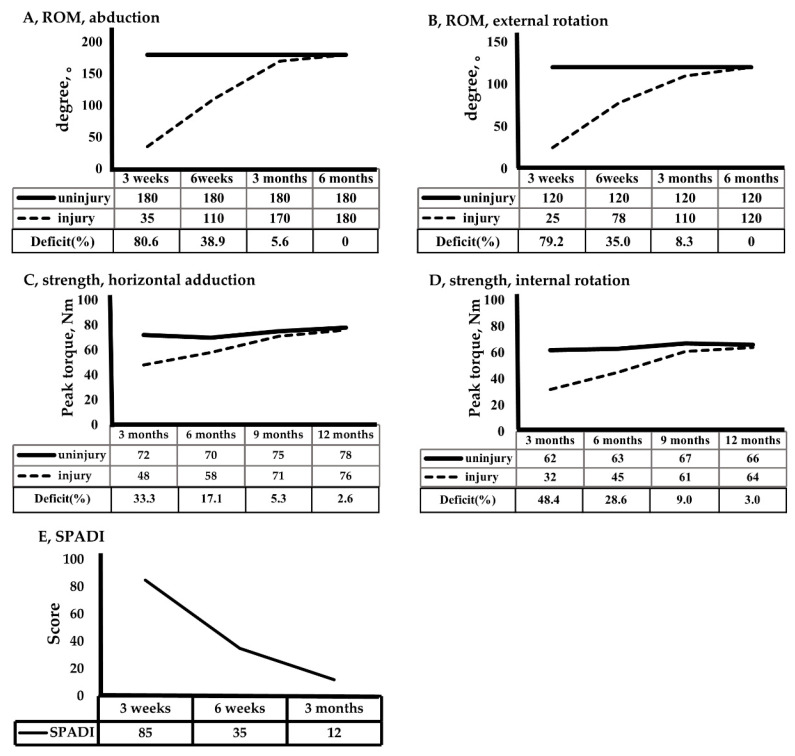
Recovery of ROM, strength, and SPADI.

ROM: Abduction has a reference value of 180°. The injured side recovered to 35° at 3 weeks and 170° at 3 months. External rotation was 25° at 3 weeks, 78° at 6 weeks, and 110° at 3 months. ROM improved linearly from 3 weeks to 3 months. Three months were required for recovery to an angle within 10%, and full ROM was obtained in 6 months (Figure 4).

Strength: Isotonic strength training using a tube band was introduced at 6 weeks, and strength tests were performed at 3, 6, 9, and 12 months. The participant started high intensity strength training at 5 months and demonstrated a noticeable improvement in muscle strength. The peak torque (Nm) and deficit (%) of horizontal adduction were 48 Nm and 33.3% at 3 months, 58 Nm and 17.1% at 6 months, and 71 Nm and 5.3% at 9 months. The deficit (%) of internal rotation was 48.4% at 3 months, 28.6% at 6 months, and 9.0% at 9 months. Recovery of horizontal abduction tended to be slightly faster than recovery of internal rotation (Figure 4).

SPADI: As a method of subjectively evaluating the patient’s shoulder condition using a questionnaire, a high score means an uncomfortable condition and a low score means a good condition. The SPADI was 85 at 3 weeks, 35 points at 6 weeks, and 12 points (no discomfort) at 3 months. The SPADI’s rate of improvement was higher 6 weeks after beginning tube strength and active assist ROM exercises than 3 weeks after surgery.

For patient’s exercise adherence, we asked the patient to record exercise performance, pain, and condition every day. We reviewed the content and provided feedback and consultation. There were no adverse and unanticipated events or problems during rehabilitation until return to sports.

## 4. Discussion

PM muscle rupture is a rare injury, but when it does occur, it is almost always during sports or exercise activities. There are some studies related to its surgical treatment, but the literature specifically about rehabilitation is insufficient [8,9,10]. Therefore, the purpose of this study was to report the results of the process of rehabilitation, offer insight into the recovery of muscle strength and ROM, and evaluate the patient’s scores over a one-year period.

One of the main features of this study is the early initiation of ROM exercises. Previous studies generally started ROM exercises at 4–6 weeks after surgery [5,8,17,18,19], and one study banned passive ROM exercise until 12 weeks [10]. However, in the literature that introduced the rehabilitation protocol after PM repair, mild passive ROM was possible at 2 weeks [11]. Based on this information, this study initiated passive ROM on 2 weeks after surgery. A difference of about 30% was observed at Week 6, and almost full ROM was recovered at 3 months. Although there are differences between the injuries, in studies regarding rotator cuff repair, pain and ROM recovery rates were better in groups with early ROM exercises after surgery than those immobilized for 6 weeks [20,21]. Early ROM exercises have several advantages. They can relieve pain, reduce swelling, and prevent stiffness caused by immobilization [22]. In our study, forward flexion with the patient in the supine position was passively performed to the shoulder level for safety, and abduction and external rotation were performed to stimulate the PM muscle with tolerated passive ROM starting at 4 weeks.

Early exercises in this study were not only ROM exercises, but also included isometric strength exercises of the PM muscle, elbow flexion and extension, wrist and scapular exercises, and shrugging. These exercises were considered “positive” exercises because they helped to prevent stiffness in the surrounding muscles, relieve pain, and safely protect the surgical site. Similar to other studies, isotonic strength training was introduced at 6 weeks, which coincides with the timing of physiological recovery [8,11]. However, strength training was also a little faster than that reported in other studies. In some studies, strength training began gradually after 12 weeks, whereas others initiated isometric contractions at 2 months [5] and isotonic resistance exercise at 3 months [19].

The most important exercise to consider in strength training is the bench press. The bench press is a major way to exercise the PM and is associated with the highest incidence of injury among PM ruptures [1,23]. As a result, the bench press was not performed until 3 months after surgery in previous studies [9]. In our study, the following strategy was designed by applying the principle of muscle neuromuscular mechanics for safe weight training [24]. The patient was instructed to use a chair chest press instead of a bench press, thereby offsetting gravity. Next, the patient practiced contracting the PM muscle with a tube band before proceeding to machine-based weight training. In addition, the patient was limited in terms of lengthening and elastic movement of the PM muscle until the ability to control the length and tension of the Golgi tendon and muscle spindle was sufficiently adapted during eccentric contraction [24,25]. Therefore, the exercise intensity was light, and the range of motion gradually increased.

The return to sports activities in this case was 5 months, and Jiu-Jitsu competition was possible at 7 months. These results were similar or slightly faster than previous studies. In most studies, return to sports occurred between 5.5 and 6 months [7,17,18]. Moreover, Judo athletes, similar to the patient in this study, returned at 6 months, but could not compete until 9 months [9]. Whether this study’s rapid recovery is due to early ROM cannot be determined, and further research is required.

As noted above, this study examined an amateur Jiu-Jitsu athlete’s PM rupture. Jiu-Jitsu is a traditional Asian martial art, and its characteristic techniques include punching, kicking, breaking joints, and knocking down an opponent. Accordingly, Jiu-Jitsu athletes often injure upper bodies more frequently than lower extremity due to Jiu-Jitsu’s punch and joint break technique [12]. In a study that analyzed 60 cases, six cases involved injury during Jiu-Jitsu, and ours is one of only a few reports of a Jiu-Jitsu-related PM rupture [17]. Although PM muscle rupture is a rare occurrence, more studies examining this injury are being reported, perhaps as a result of increasing participation in weight training, contact sports, and mixed martial arts [5,17,26]. When the mechanism of injury was classified, 28 out of 693 cases occurred in wrestling, boxing, and judo—similar types of exercise to Jiu-Jitsu [27].

One of the key features of this study was its measurement of muscle strength using isokinetic equipment four times every 3 months. Prior to this study, several studies had already explored the isokinetic assessment of PM rupture patients. Most were performed to compare the effects of surgical and non-surgical treatments, and the results revealed that patients who received surgical treatment showed higher peak torque than those in the non-surgical treatment group [17,18,19,28]. Recall that previous studies showed that the surgical group recovered up to 99% of strength, while the non-surgical group only regained up to 56% [7]. In another study, a surgical group exhibited a strength difference of 14.3%, while the non-surgical group exhibited a difference of 41.7% [17]. In this study, muscle recovery regained up to a difference of 17.1% in horizontal pronation and 22.2% in internal rotation at 6 months after surgery. The normal range of isokinetic strength deficit was 10% in the measurement manual [14], and “good” recovery was 20% [17]. Applying this criterion, the patient in the current study required about 6 months to recover to “good” and 9 months to “normal” range. However, since previous studies only conducted one test, understanding the change over time was not possible, thereby making it difficult to compare those results with the results of this study. One possible reason for this discrepancy is that PM rupture is very rare, so there are limited opportunities to apply standard protocols of measurement. The timing of the strength test would have varied from 2 months to 108 months [7].

In addition to maximal muscle strength, surgical treatment exhibited better results than non-surgical treatment. In the evaluation using the Clinical Bak Functional Criteria, 67% of surgical patients recovered to excellent status, 22.6% to good, and 0% to fair, but 0% of non-surgical patients recovered to excellent, 27.6% to good, 41.4% to fair, and 31.07% to poor [17]. Patients’ subjective satisfaction was 96% in the surgical group, but 51% in the non-surgical treatment group [19]. Therefore, surgery is recommended for relatively active individuals, and conservative therapy is an option for the elderly, individuals who refuse surgery, and those with only partial tears [10,29]. The condition and location of the rupture also helps determine whether surgery is necessary. Of the 365 cases of PM rupture, 245 were reported to occur at musculotendinous junctions. It should be noted that the location is the tendon close to the insertion of the humerus and that a complete rupture of the tendon is difficult to regenerate without surgical intervention [26]. However, in a meta-analysis that analyzed 664 cases from 23 surgical-related studies, surgical complications occurred in 14.21% of cases; specifically, re-rupture occurred in 3.08%, additional surgery was necessary in 2.28%, and persistent pain occurred in 3.03% of cases [30]. This information should be considered when choosing surgical treatment.

This study presented the order of muscle strength recovery. The recovery of horizontal pronation was faster than that of internal rotation. The possibilities of such recovery will differ depending on the surrounding muscle relationships. Because adduction occurs due to the cooperation of the anterior deltoid muscle, it can disperse the force of the PM muscle, whereas internal rotation can be relatively difficult under maximum force because it has to rely almost exclusively on the PM [31]. PM muscle rupture occurs mostly during exercise and sports activities, and a successful return to sports activity after surgery is the ultimate goal of patients, doctors, and therapists.

The strength of this study is that early ROM and muscle strength interventions were applied after PM rupture, and the rehabilitation process and various evaluations over time were recorded in detail for one year. Nevertheless, this study’s method had some limitations. First, this study used only isokinetic equipment to measure muscle strength. Because this equipment is expensive, there is a limit to its popularization. As an alternative test method, there is a handheld dynamometer, which is relatively inexpensive, has high validity, portability, and a simple measurement method [32,33]. Next, functional tests such as the Y-balance test were not tested. Although this test has been widely applied as a dynamic lower limb balance test, several studies have shown that it is a useful tool for evaluating the functionality of the upper limb test [34,35].

An additional limitation is the rehabilitation program. It is difficult to argue that this study has unique characteristics compared to existing musculoskeletal rehabilitation studies, except for its early application of passive ROM exercises. However, since PM rupture is a very rare injury, there are relatively few rehabilitation studies that can be referenced. We chose the routine rehabilitation method because we prioritized safety.

Since our study is a case study and not an evidence-based practice, there is a limit to generalizing the results of this study. Future research will require the establishment of popular rehabilitation protocol and experimental study.

## 5. Conclusions

As a result of applying ROM and strength training after the pectoral rupture repair surgery, ROM and function score recovery were achieved at 3 months post-surgery and muscle recovery at 6 months. Return-to-sport was possible at 5 months without complications and side effects, and competition was possible at 7 months. Therefore, for this particular patient, rehabilitation after pectoral rupture repair surgery was effective in restoring function and strength and facilitating return to sports.

## Figures and Tables

**Table 1:** Summary of rehabilitation exercises based on recovery time.

Post Operation	Exercise
At 1 week	Absolute immobilization Wound protection and application of sling Hand grip: 10 rep × 3 set, 2 times/day Shrugging: 10 rep × 3 set, 2 times/day Scapular mobilization: 10 rep × 3 set, 2 times/day
At 2–3 weeks	Early passive ROM exercise Passive ROM; forward flexion to 90°: 5 s × 6 set, 3 times/day Pendulum exercise; forward and backward: 20 rep × 2 set, 3 times/day
At 4–5 weeks	Aggressive passive ROM and isometric contraction Passive ROM; forward flexion above 90° with T-bar or pulley: 5 s × 6 set, 5 times/day Passive ROM; external rotation and abduction: 5 s × 6 set, 5 times/day Isometric contraction of chest and upper body: 20 rep × 2 set, 3 times v Pendulum exercise; multi-direction: 20 rep × 2 set, 3 times/day
After 6 weeks	Active assist ROM and isotonic contraction Active assist ROM; external rotation, flexion, abduction: 10 s × 6 set, 5 times/day Isotonic contraction with tube band; abduction, internal rotation: 10 rep × 2 set, 2 times/day
After 12 weeks	Mile machine weight training; chest press Chest press within 60° and low to moderate intensity: 15 rep × 3 set, 2 times/day Adduction and internal rotation strength with tube band: 20 rep × 3 set, 3 times/day Deltoid and biceps strength exercise with machine: 20 rep × 3 set, 2 times/day
After 5 months	Aggressive bench press with high intensity and neuromuscular training Bench press with high intensity: 20 rep × 3 set, 1 times/day Above 8 items weight training: 20 rep × 3 set, 1 times/day Push up on the unstable tool such as foam roller and gym ball: 20 rep × 3 set, 2 times/day Preparing return to sports
After 6 months	Power training for return to sports Power training; adduction and internal rotation: high intensity, 2 times/day Plyometric training with medicine-ball, trampoline: high intensity, 2 times/day

Abbreviation: ROM, range of motion; rep, repetition; s, seconds.

## Data Availability

The data are not publicly available due to privacy or ethical.

## References

[1] Butt U., Mehta S., Funk L., Monga P. (2015). Pectoralis major ruptures: A review of current management. J. Shoulder Elb. Surg..

[2] Boudreau N., Gaudreault N., Roy J.-S., Bédard S., Balg F. (2019). The addition of glenohumeral adductor coactivation to a rotator cuff exercise program for rotator cuff tendinopathy: A single-blind randomized controlled trial. J. Orthop. Sports Phys. Ther..

[3] Thompson K., Kwon Y., Flatow E., Jazrawi L., Strauss E., Alaia M. (2020). Everything pectoralis major: From repair to transfer. Physician Sportsmed..

[4] Yu J., Zhang C., Horner N., Ayeni O.R., Leroux T., Alolabi B., Khan M. (2019). Outcomes and return to sport after pectoralis major tendon repair: A systematic review. Sports Health.

[5] Cordasco F.A., Mahony G.T., Tsouris N., Degen R.M. (2017). Pectoralis major tendon tears: Functional outcomes and return to sport in a consecutive series of 40 athletes. J. Shoulder Elb. Surg..

[6] Chang E.S., Zou J., Costello J.M., Lin A. (2016). Accuracy of magnetic resonance imaging in predicting the intraoperative tear characteristics of pectoralis major ruptures. J. Shoulder Elb. Surg..

[7] Hanna C., Glenny A., Stanley S., Caughey M. (2001). Pectoralis major tears: Comparison of surgical and conservative treatment. Br. J. Sports Med..

[8] Vasiliadis A.V., Lampridis V., Georgiannos D., Bisbinas I.G. (2016). Rehabilitation exercise program after surgical treatment of pectoralis major rupture. A case report. Phys. Ther. Sport.

[9] Hoppes C.W., Ross M.D., Moore J.H. (2013). Undetected pectoralis major tendon rupture in a patient referred to a physical therapist in a combat environment: A case report. Phys. Ther..

[10] Shepard N.P., Westrick R.B., Owens B.D., Johnson M.R. (2013). Bony avulsion injury of the pectoralis major in a 19 year-old male judo athlete: A case report. Int. J. Sports Phys. Ther..

[11] Manske R.C., Prohaska D. (2007). Pectoralis major tendon repair post surgical rehabilitation. N. Am. J. Sports Phys. Ther. NAJSPT.

[12] Scoggin J.F., Brusovanik G., Izuka B.H., Zandee van Rilland E., Geling O., Tokumura S. (2014). Assessment of injuries during Brazilian jiu-jitsu competition. Orthop. J. Sports Med..

[13] Keteyian S.J. (2004). ACSM Certification: ACSM Certification Update: Standards and Guidelines for Academic Programs and the New Certification for Personal Trainers. Acsm’s Health Fit. J..

[14] CSMi (2019). Humac Norm Users Guide.

[15] Dvir Z. (2004). Isokinetics: Muscle Testing, Interpretation, and Clinical Applications.

[16] Williams J.W., Holleman D.R., Simel D. (1995). Measuring shoulder function with the Shoulder Pain and Disability Index. J. Rheumatol..

[17] de Castro Pochini A., Andreoli C.V., Belangero P.S., Figueiredo E.A., Terra B.B., Cohen C., Andrade M.d.S., Cohen M., Ejnisman B. (2014). Clinical considerations for the surgical treatment of pectoralis major muscle ruptures based on 60 cases: A prospective study and literature review. Am. J. Sports Med..

[18] Kang R.W., Mahony G.T., Cordasco F.A. (2014). Pectoralis major repair with cortical button technique. Arthrosc. Tech..

[19] Schepsis A.A., Grafe M.W., Jones H.P., Lemos M.J. (2000). Rupture of the pectoralis major muscle: Outcome after repair of acute and chronic injuries. Am. J. Sports Med..

[20] Keener J.D., Galatz L.M., Stobbs-Cucchi G., Patton R., Yamaguchi K. (2014). Rehabilitation following arthroscopic rotator cuff repair: A prospective randomized trial of immobilization compared with early motion. JBJS.

[21] Arndt J., Clavert P., Mielcarek P., Bouchaib J., Meyer N., Kempf J.-F., the French Society for Shoulder & Elbow (SOFEC) (2012). Immediate passive motion versus immobilization after endoscopic supraspinatus tendon repair: A prospective randomized study. Orthop. Traumatol. Surg. Res..

[22] Chang K.-V., Hung C.-Y., Han D.-S., Chen W.-S., Wang T.-G., Chien K.-L. (2015). Early versus delayed passive range of motion exercise for arthroscopic rotator cuff repair: A meta-analysis of randomized controlled trials. Am. J. Sports Med..

[23] Bak K., Cameron E., Henderson I. (2000). Rupture of the pectoralis major: A meta-analysis of 112 cases. Knee Surg. Sports Traumatol. Arthrosc..

[24] Chalmers G. (2002). Strength training: Do Golgi tendon organs really inhibit muscle activity at high force levels to save muscles from injury, and adapt with strength training?. Sports Biomech..

[25] LaStayo P.C., Woolf J.M., Lewek M.D., Snyder-Mackler L., Reich T., Lindstedt S.L. (2003). Eccentric muscle contractions: Their contribution to injury, prevention, rehabilitation, and sport. J. Orthop. Sports Phys. Ther..

[26] ElMaraghy A.W., Devereaux M.W. (2012). A systematic review and comprehensive classification of pectoralis major tears. J. Shoulder Elb. Surg..

[27] Bodendorfer B.M., McCormick B.P., Wang D.X., Looney A.M., Conroy C.M., Fryar C.M., Kotler J.A., Ferris W.J., Postma W.F., Chang E.S. (2020). Treatment of pectoralis major tendon tears: A systematic review and meta-analysis of operative and nonoperative treatment. Orthop. J. Sports Med..

[28] Fleury A.M., Silva A.C.d., Pochini A.A.d.C., Ejnisman B., Lira C.A.B.d., Andrade M.d.S. (2011). Isokinetic muscle assessment after treatment of pectoralis major muscle rupture using surgical or non-surgical procedures. Clinics.

[29] Zvijac J.E., Schurhoff M.R., Hechtman K.S., Uribe J.W. (2006). Pectoralis major tears: Correlation of magnetic resonance imaging and treatment strategies. Am. J. Sports Med..

[30] Bodendorfer B.M., Wang D.X., McCormick B.P., Looney A.M., Conroy C.M., Fryar C.M., Kotler J.A., Ferris W.J., Postma W.F., Chang E.S. (2020). Treatment of pectoralis major tendon tears: A systematic review and meta-analysis of repair timing and fixation methods. Am. J. Sports Med..

[31] Wolfe S.W., Wickiewicz T.L., Cavanaugh J.T. (1992). Ruptures of the pectoralis major muscle: An anatomic and clinical analysis. Am. J. Sports Med..

[32] Johansson F.R., Skillgate E., Lapauw M.L., Clijmans D., Deneulin V.P., Palmans T., Engineer H.K., Cools A.M. (2015). Measuring eccentric strength of the shoulder external rotators using a handheld dynamometer: Reliability and validity. J. Athl. Train..

[33] Le-Ngoc L., Janssen J. (2012). Validity and reliability of a hand-held dynamometer for dynamic muscle strength assessment. Rehabil. Med..

[34] Wassinger C.A., McKinney H., Roane S., Davenport M.J., Owens B., Breese U., Sokell G.A. (2014). The influence of upper body fatigue on dynamic standing balance. Int. J. Sports Phys. Ther..

[35] Borms D., Cools A. (2018). Upper-extremity functional performance tests: Reference values for overhead athletes. Int. J. Sports Med..

